# The German AugUR study: study protocol of a prospective study to investigate chronic diseases in the elderly

**DOI:** 10.1186/s12877-015-0122-0

**Published:** 2015-10-21

**Authors:** Klaus Stark, Matthias Olden, Caroline Brandl, Alexander Dietl, Martina E. Zimmermann, Sabine C. Schelter, Julika Loss, Michael F. Leitzmann, Carsten A. Böger, Andreas Luchner, Florian Kronenberg, Horst Helbig, Bernhard H. F. Weber, Iris M. Heid

**Affiliations:** Department of Genetic Epidemiology, University Regensburg, Franz-Josef-Strauss-Allee 11, 93053 Regensburg, Germany; Department of Ophthalmology, University Hospital Regensburg, Regensburg, Germany; Institute of Human Genetics, University Regensburg, Regensburg, Germany; Department of Internal Medicine II, University Hospital Regensburg, Regensburg, Germany; Centre for Clinical Studies, University Hospital Regensburg, Regensburg, Germany; Department of Epidemiology and Preventive Medicine, University Regensburg, Regensburg, Germany; Department of Nephrology, University Hospital Regensburg, Regensburg, Germany; Division of Genetic Epidemiology, Medical University of Innsbruck, Innsbruck, Austria

**Keywords:** Mobile elderly population, Cross-sectional study, Cohort study, Etiology of chronic diseases, Diseases of late onset, Study platform, Genetic and non-genetic risk factors

## Abstract

**Background:**

The majority of patients suffering from chronic health disabilities is beyond 70 years of age. Typical late-onset chronic diseases include those affecting the heart, the kidney, cancer, and conditions of the eye such as age-related macular degeneration. These diseases disable patients for many years and largely compromise autonomy in daily life. Due to challenges in recruiting the elderly, the collection of population-based epidemiological data as a prerequisite to understand associated risk factors and mechanisms is commonly done in the general population within an age-range of 20 to 70 years.

**Methods/Design:**

We establish the German AugUR study (*A*ge-related diseases: *u*nderstanding *g*enetic and non-genetic influences - a study at the *U*niversity of *R*egensburg), a prospective study in the mobile elderly general population in and around Regensburg in eastern Bavaria. In the long term, we aim to recruit 3,000 persons of Caucasian ethnicity with at least 70 years of age via residents’ registration offices and conduct 3-year follow-ups.

The study protocol includes a standardized interview regarding social and life-style factors, medication history, quality-of-life, and existing diagnoses of common diseases. The participants undergo medical examinations for ophthalmological, cardiovascular or diabetes-related conditions, and general measurements of body shape and fitness. The program is particularly tailored for the elderly. Biobanking of whole blood, serum, plasma, and urine is conducted and standard laboratory measurements are performed in fresh samples.

**Discussion:**

AugUR is specifically designed as a research platform to host studies of late onset diseases. Consequently, this platform will help (1) to unravel the genetic and non-genetic etiology of disease development and progression, (2) to serve as control group of elderly individuals for comparisons with various patient groups, (3) to derive prevalence and incidence data on chronic diseases, and (4) to provide clinical reference parameters for the elderly mobile general population. This data will foster our understanding of disease mechanisms, which may ultimately help to improve prevention, diagnosis, and therapy for frequent chronic diseases. Here we present the baseline study protocol of AugUR.

## Background

A major proportion of hospitalized patients is above 70 years of age. This is mainly due to a variety of chronic diseases that tend to occur later in life and disable the elderly patients for many years or even decades. Such diseases include type 2 diabetes [1], cancer [2], cardiovascular complications [3], and eye diseases like age-related macular degeneration (AMD) [4], the leading cause of blindness in the elderly.

Morbidity and disability at high age do not only affect individuals’ quality of life, but also impact upon public health care systems. Such challenges will increase given demographic changes towards a growing proportion of persons above 80 years of age, in Europe from currently 4 to 10 % in 2050 [5]. Consequently, the number of patients with late-onset diseases will increase. Public health efforts that help increase life expectancy also need to include measures to avoid disabling diseases.

To understand the etiology of late-onset diseases, it is important to know their genetic architecture. Most diseases have a genetic component with a heritability of 40–70 % [6, 7] and numerous genetic loci identified [8, 9]. The knowledge of the genetic background provides insights to disease mechanisms and will stimulate ideas for new therapeutic options. Genome-wide association studies (GWAS) have been immensely successful in describing the genetic basis for numerous diseases [8]. To increase statistical power and to identify even small genetic effects, such analyses are conducted typically by combining the results of multiple studies in meta-analyses. Previous results from GWAS have usually been obtained by merging studies of diverse designs, often recruited from patient groups with limited assessment of non-genetic risk factors, and lack of follow-up. In order to investigate the combination of genetic and life style factors in relation to disease incidence and progression, large population-based prospective follow-up studies with high quality phenotyping are warranted.

Current large-scale prospective epidemiologic studies often focus on adults less than 70 years of age, such as the German National Cohort [10] or the UK Biobank [11]. This is due to the fact that recruiting elderly individuals is particularly challenging regarding the time and patience required for each step of the study program, including walking from one room to another, un- and re-dressing, or answering questions. A further challenge is the impaired visual, auditory, or cognitive function frequently encountered in elderly individuals, necessitating a study program that is tailored to their specific conditions.

Therefore, we set out to establish a study platform using population-based recruitment of mobile elderly individuals in and around the east Bavarian city of Regensburg. This platform will allow for harboring studies on various late-onset common diseases using cross-sectional, case-control, or cohort study designs.

## Methods/design

### Main objective and overall design

The main objective of our prospective study platform is to build a data base that enables the investigation of genetic and non-genetic risk factors as well as biomarkers for late-onset diseases. The AugUR study (*A*ge-related diseases: *u*nderstanding *g*enetic and non-genetic influences - a study at the *U*niversity of *R*egensburg) is a prospective study from the general elderly population in and around the city of Regensburg. The baseline survey includes an in-person interview, biobanking and medical examinations. A follow-up with a second in-person visit after 3 years and subsequent mortality and morbidity follow-ups are planned.

### Study population and recruitment

Our study cohort consists of the mobile elderly population at least 70 years of age. The recruitment area is the city and county of Regensburg, which is a middle-sized Bavarian city with ~150,000 inhabitants located in the South-East of Germany with the next bigger cities >100 km away; the study region includes both urban and rural areas with a total of ~330,000 inhabitants, mostly of Caucasian ethnicity. We are aiming at 3,000 participants during a five year recruitment period from 2013 to 2018 with subsequent follow-ups.

For the baseline survey, we obtain a random sample from the local registries of residence. Selected inhabitants are contacted by a mailed invitation letter giving the choice to answer per phone or via a pre-paid post card stating their phone number. Those that contact the study center in order to raise their unwillingness to participate are asked to complete a short phone interview to assess the reasons for refusal, if possible. These persons are not contacted again. In addition, a random subset of non-responders undergoes a non-compliance interview by phone.

Persons indicating their interest in participating the study are contacted and an appointment is made. There are no a priori exclusion criteria. Invited persons are welcomed at the recruitment center at the University Hospital Regensburg. Informed consent is obtained by trained study staff, and those giving consent are subjected to the study program. Participants are asked for agreement to be re-contacted for follow-ups.

### Ethic approval, data protection, and informed consent

The study protocol, study procedures, and data protection strategy were all approved by the Ethics Committee of the University of Regensburg, Germany (vote 12-101-0258). Written consent is obtained according to the Declaration of Helsinki. Person-identifying information is strictly separated from the study results using different computer systems and identifying numbers. Original data are stored in a physically safe place with encrypted backup and access limited to a restricted group of staff to ensure safety and confidentiality. Each participant is specifically asked for consent to be contacted in the future, to allow for genetic analyses, and whether he/she wishes feedback regarding their results from the physical and medical examinations and the standard laboratory panel.

### Overview of the questionnaire and the physical and medical examinations

An in-person standardized interview and physical and medical examinations are conducted by trained staff. The questionnaire includes an assessment of socio-demographic (marital status, education, profession) and life style factors (nutrition, dietary supplements, smoking behavior, alcohol consumption, physical activity), comorbidities, medication use, and quality of life. The physical and medical examinations consist of an ophthalmological and a cardiovascular program, measurement of diabetes-related parameters, general fitness and anthropometry (Table 1). Biobanking is conducted for aliquoted serum, plasma, blood for DNA and RNA as well as urine (Table 2).

**Table 1:** Baseline assessment in AugUR

Category	Item	Instrument	Method/apparatus used
General factors	Age, sex, ethnicity, martial status	Q	Interview
	Education, profession	Q	Interview
	Nutritional habits	Q	Interview
	Smoking, alcohol consumption	Q	Interview
	Light exposure	Q	Interview
Quality of life	Subjective health status, general condition	Q	Interview
Anthropometry	Height, weight	P	Measuring station
and fitness	Waist and hip circumferences	P	Measuring tape
	Hand grip strength	P	Dynamometer
	Physical activity	Q	Interview
Medication	Current medication intake	Q	Interview
Ophthalmological	Eye diseases and eye injuries	Q/P	Interview, DRS, OCT
Phenotyping	Ocular fundus	P	DRS, OCT
	Visual acuity	P	ETDRS, Amsler
Cardiovascular	Cardiovascular diseases	Q	Interview
Phenotyping	Blood pressure, pulse rate	P	Automatic device
	Ankle-brachial index	P	Vascular Explorer
	Aortic pulse wave velocity	P	Vascular Explorer
	Augmentation index	P	Vascular Explorer
	Left/right ventricular mass and function	P	Echocardiography
	Assessment of heart valves	P	Echocardiography
Diabetes	History of diabetes, diabetes medications	Q	Interview
	Skin autofluorescence	P	AGE reader
	Serum glucose, HbA_1c_	L	External laboratory
Blood parameters	Blood cell count	L	External laboratory
	Mean corpuscular cell volume	L	External laboratory
	Mean corpuscular hemoglobin content	L	External laboratory
	Mean cellular hemoglobin concentration	L	External laboratory
	C-reactive protein	L	External laboratory
	LDL-, HDL-, total cholesterol, triglycerides	L	External laboratory
Urine parameters	Albumin:Creatinine, Protein:Creatinine ratio	L	Clinitek
	Glucose, ketone, nitrite, pH	L	Clinitek
	Erythrocyte and leukocyte count	L	Clinitek
Other diseases	Respiratory tract diseases	Q	Interview
	Kidney diseases	Q	Interview
	Arthritic diseases	Q	Interview
	Skin diseases	Q	Interview
	Liver diseases	Q	Interview
	Cancer	Q	Interview

*Q *self-reported information obtained by questionnaire, *L* laboratory analysis, *P* physical examination, *HBA*_*1c*_ glycated hemoglobin, *LDL* low-density lipoprotein, *HDL* high-density lipoprotein, *AGE* advanced glycation end products, *DRS* digital retinography system, *OCT* optical coherence tomography, *ETDRS* early treatment diabetic retinopathy study

**Table 2:** Biobanking in AugUR

Origin	Fraction	Number of aliquots	Storage conditions
K3-EDTA	Whole blood	6 + 4	−20 °C/-80 °C
K3-EDTA	Plasma	12 + 2	−80 °C/Liquid N_2_
Clot activator	Serum	12 + 2	−80 °C/Liquid N_2_
PAXgene	RNA supernatant	2	Liquid N_2_
PAXgene	RNA cellular fraction	1	Liquid N_2_
Urine	Urine supernatant	1	Liquid N_2_
Urine	Urine with pellet	2	−80 °C

Questionnaire data and documentation of examinations are initially recorded on paper and are subsequently transferred to an electronic case report form (eCRF). We opted against using an ad hoc eCRF during the interview, as we found that interaction with elderly persons requires full eye contact and parallel entering of information into the computer system did not yield a time benefit, but caused a disruption of the interview. For collecting and managing eCRF data, a specialized software tool is employed (Askimed, http://www.askimed.com/). Quality control includes monitoring the entered data by a second independent study assistant.

### Ophthalmological examination program

#### Overview

Interview-based ophthalmological medical history assesses the existence, history and time of onset of major eye diseases (cataract, glaucoma, diabetic retinopathy, AMD, former ocular injury or severe infection, family history of eye diseases) or ophthalmological interventions (eye surgery, intravitreal injections, laser treatment, application of eye drops or other eye-specific medication).

#### Amsler grid test and visual acuity

In order to assess irregularities in central vision, an *Amsler grid test* is conducted according to [12]. Briefly, participants are tested, each eye separately, with a black-on-white Amsler grid (10×10 cm with a 0.5-cm grid) at a reading distance of 30 cm using their most recent reading glasses if needed and available. Participants are instructed to fixate the black spot in the center. The test is classified as positive when the participant claims to see irregularities in the grid (interruptions in the network of the small squares; lines are not straight and parallel; vibration or waving areas; gray hazed areas).

*Visual acuity* is the main endpoint for ophthalmological diseases. A rapid and simplified visual acuity test is carried out on a standardized Early Treatment Diabetic Retinopathy Study (ETDRS)-chart for the lines with equivalence values of 20/25 Snellen Equivalent and 20/20 Snellen Equivalent at a distance of 4 m. Study participants use their most recent distance glasses if applicable. When ≥60 % letters are read correctly, the visual acuity score is increased by +1 at 20/25 or +2 at 20/20, respectively, for each eye separately. When <60 % letters of 20/25 equivalence value are read correctly, the visual acuity score per eye is -2.

#### Photo stress test

Dark or white adaptation time, which is the time of full adjustment and reading ability of the eye after darkness or very bright light, respectively, provide a psychophysical assessment of retinal function [13–15]. These parameters are associated with multiple eye diseases and are used as surrogate endpoints in clinical studies [16]. While dark adaptation requires high attention and patience of the study participants, the photo stress test to assess white adaptation can be better applied in a standardized fashion. This strategy is the most appropriate and clinically applicable photo-stress bleaching technique, as high intensity, long-duration bleaching minimizes errors [17].

Briefly, starting with the eye with better visual acuity, the participant´s refractive correction (if applicable) is removed and the participant is administered to the macular stop of a direct ophthalmoscope (Heine Beta 200 M2, Heine Optotechnik, Herrsching, Germany). The ophthalmoscope is adjusted to full intensity, projecting directly onto the macula for 30 s. Immediately after bleaching, the time until the participant can read one line less than before in the visual acuity test is measured in seconds with refractive correction (if applicable).

#### Fundus camera imaging

Many eye diseases affect the retina. We thus conduct fundus photography using the automatized DRS fundus camera (Digital Retinography System; CenterVue, Padova, Italy) [18]. Pictures are captured for the central and central nasal field of the retina within a 45° view. The quality of the fundus photographs depends on the pupil size. Therefore, participants´ eyes are allowed to adapt in a darkened room for 5 min before photography. Since pupil size is known to be age-dependent [19], it is likely that pupils in our elderly participants are often too small to yield a high-quality photography. Therefore, we apply a mild mydriasis (Mydriaticum UD, pharmaSTULLN, Stulln, Germany), when special consent is given. Participants are explicitly informed about the consequences of mydriasis, such as a ban on driving, and a small risk for acute angle-closure glaucoma (1 in 20,000 to 1 in 3,000) [20, 21]. This risk is minimized by excluding persons from mydriasis that exhibit a flat anterior chamber, as assessed via a portable slit lamp examination (Kowa, Düsseldorf, Germany).

#### Optical coherence tomography

Comparable to ultra-sound examinations of inner organs, the optical coherence tomography (OCT) scans the retina, especially the macula and the optic disc using light. We apply confocal scanning laser ophthalmoscopy (cSLO) and spectral domain OCT (SD-OCT) using the SPECTRALIS OCT Plus BluePeak (Heidelberg Engineering GmbH, Heidelberg, Germany). This includes several views of the retina: (i) a set of *high-resolution cSLO fundus images* including infrared, blue reflectance, BluePeak autofluorescence as well as multicolor mode for each participants’ eye in 30° (8.7 mm) standard fovea centered view field with 30 averaged frames by Automatic Real-Time (ART), (ii) a set of *fovea-centered SD-OCT scans* (all in standard TruTrack active eye tracking mode and with quality index as high as possible) including one 30° (8.7 mm) horizontal scan averaged with 100 ART, and horizontal raster pattern scans, 20°x20° (5.8 mm x 5.8 mm), consisting of 49 sections, each averaged with 30 ART, and, additionally one 30° (8.7 mm) horizontal and vertical line, each averaged with 100 ART in enhanced depth imaging mode, and (iii) a *circled SD-OCT scan around the optic disc* with 100 ART to assess retinal nerve fiber layers.

SD-OCT scans and cSLO images are analyzed applying the Heidelberg Eye Explorer software (Heidelberg Engineering GmbH, Heidelberg, Germany).

### Cardiovascular examinations

#### Overview

The interview-based medical history assessment includes the existence, history, and time of onset of cardiovascular diseases and interventions. Lipid parameters are measured in fresh blood in non-fasting condition. Fasting before study center visit was unacceptable for our elderly study population. In order to assess subclinical and clinical cardiovascular conditions, we established an in-depth phenotyping program including measurement of blood pressure, pulse rate, vascular status, and imaging by a short (for all) and an extended (for a subgroup) cardiac ultrasound protocol as described below.

#### Blood pressure, pulse and vascular phenotyping

Blood pressure and pulse rate are measured using an automatic device (Omron M10-IT; Omron Healthcare Co Ltd., Kyoto, Japan) after 5 min of resting time. Blood pressure is measured three times and the average of the second and third measurement is determined. Ankle-brachial index, aortic pulse wave velocity and augmentation index are derived from the Vascular Explorer system (Enverdis GmbH medical solutions, Jena, Germany).

#### Echocardiography

The echocardiographic measurements are intended to provide established and reliable parameters of the morphology and function of the heart chambers and valves. We use a commercially available ultrasound unit (HP Sonos 5500 with 2–4 MHz probe; Philips, Eindhoven, The Netherlands) and implement the recommendations of the European Association of Echocardiography for clinical trials [22]. Each measurement is repeated three to ten times according to protocol to reduce random error. The stored tracings are assessed post hoc using analytical software Xcelera R3.2 L1 Version 3.2.1.520 – 2011 (Philips, Eindhoven, The Netherlands). We established a short and an extended protocol.

The *short echocardiographic program* focuses on left ventricular function and the morphology of the four heart chambers accounting for chamber-specific cardiac remodeling processes [23]. Cardiac morphology including areas and volumes of the four heart chambers determined in the apical four- and two-chamber views as well as left ventricular dimensions assessed by two-dimensional guided M-mode in the left parasternal long axis view are recorded [24]. The left ventricular mass is calculated by the Devereux formula [25]. For functional evaluation, left ventricular systolic function is determined by the ejection fraction estimated by the modified Simpson’s method [24]. Diastolic function is assessed by pulse wave Doppler measurement of the transmitral flow profile and pulsed tissue Doppler assessment of the diastolic mitral annular velocity as described previously [26] and recommended by the American Society of Echocardiography [27]. This program is performed by a study nurse who was trained at the Cardiology Clinic of the University Hospital Regensburg and has conducted more than 300 echocardiographic examinations before starting to examine without supervision.

For a subgroup of participants, we conduct an *extended echocardiographic program* additionally assessing right ventricular function and the heart valves performed by an experienced cardiology-trained physician. The following parameters are determined: right ventricular function is estimated by tricuspid annular plane systolic excursion and the Tei-Index calculated by tissue Doppler imaging [28]. For an assessment of the heart valves, transvalvular flow profiles are acquired [29].

### Assessment of diabetes-related parameters, anthropometry and general fitness

Information about previous diagnosis of diabetes and diabetes therapy are obtained at the interview. HbA_1c_ and glucose measurements are conducted in fresh blood. In order to derive a marker for diabetes and cardiovascular risk [30], we assess skin autofluorescence to measure advanced glycation end products (AGE) using the AGE Reader SU (DiagnOptics, Groningen, The Netherlands).

Anthropometric measurements are carried out following the World Health Organization recommendations [31]. Height and weight (using a Seca measuring station 285; Seca, Hamburg, Germany) as well as waist and hip circumference (using a measuring tape) are measured in standing position with light clothing to the nearest 0.5 cm (height, waist and hip circumference) and the nearest 0.1 kg (weight).

General fitness is assessed using hand grip strength (Jamar Plus + Digital Hand Dynamometer; Patterson Medical, Warrenville, IL, USA). Each hand is tested three times using the right hand first. This test was chosen over other available fitness (e.g. 6 min walk test), since this can be conducted irrespective of visual or motor impairment, which is highly prevalent given at the age range of our participants.

Measurements of AGE, anthropometry, and hand grip strength are performed according to the protocols for the German National Cohort [10].

### Other diseases, current and recent medication intake

Previous diagnoses of other diseases are assessed at the interview. These include liver, kidney, arthritic and skin diseases as well as cancer and diseases of the respiratory tract. Current and recent medication use is assessed using IDOM software [32], directly from the pharmaceutical registration number, if available. Medication use can help pinpoint a multitude of conditions such as hyperlipidemia, hypertension, and diabetes.

We ask all participants for permission to contact their primary physician, their ophthalmologist, and the last hospital they visited. This allows for confirming and extending interview-based information on disease diagnoses. As one of two tertiary care facilities in the recruitment area, and the only one for Ophthalmology and Dermatology, the University Hospital Regensburg is the hospital that best facilitates the gathering of such information.

### Biosampling and immediate laboratory analyses

Collection and procession of biosamples are conducted following standard operation procedures developed for this study based on established methods and recommendations [33]. Deviations from these standard operation procedures (e.g. extended sample handling at room temperature) are recorded and linked with the biosample information. All samples are processed immediately and are kept on dry ice before final storage at the end of the day. Identification, assignment and link to eCRF data for biosamples including 2D-barcoded tubes are managed by self-developed integrated software. For an overview of biosample aliquots and storage see Table 2.

Non-fasting blood samples are drawn in a sitting position after at least five minutes of resting. Mild venous stasis is applied for a maximum duration of one minute. Whole blood is taken using a 21G multifly needle (Sarstedt, Nümbrecht, Germany) and is transferred into four serum tubes with clot activator, five tubes containing tripotassium ethylenediaminetetraacetate (K3-EDTA) as an anticoagulant (all Sarstedt) and one PAXgene Blood RNA tube (PreAnalytiX, Hombrechtikon, Switzerland) with a defined order (serum - > K3-EDTA - > PAXgene). For the PAXgene tube, luer (BD Biosciences, Heidelberg, Germany) and membrane adapter (Sarstedt) are applied. K3-EDTA tubes are gently mixed after blood draw for 5 min.

One K3-EDTA and one serum tube is used for immediate analysis of blood parameters in a central laboratory (synlab, Regensburg, Germany). From the other four K3-EDTA tubes, a total of four aliquots à 750 μl are transferred to 2D-barcoded tubes (ABgene or Matrix, Thermo Scientific, Hudson, NH, USA) for storage at -80 °C. For subsequent DNA isolation, 6 aliquots à 1.5 ml are frozen at -20 °C in 2 ml standard tubes (Sarstedt). The remaining K3-EDTA tubes are immediately centrifuged at 2,000 g for 15 min at room temperature to separate plasma from the cellular fraction. Supernatants are transferred to a fresh 15 ml tube (Sarstedt) and mixed carefully by pipetting up and down. Twelve plasma aliquots à 500 μl are transferred to 2D-barcoded tubes (Thermo Scientific) for storage at -80 °C. Additionally, two aliquots à 1.5 ml are stored in sterile 2 ml cryo tubes (Sarstedt) in liquid nitrogen gas phase.

Serum tubes with clot activator are left in upright position for 30 min after blood draw and are centrifuged at 2,000 g for 15 min at room temperature to separate serum from the cellular fraction as soon as possible. Supernatants from three serum tubes are transferred to a fresh 15 ml tube (Sarstedt) and mixed carefully by pipetting up and down. Twelve serum aliquots à 500 μl are transferred to 2D-barcoded tubes (Thermo Scientific) for storage at -80 °C. Additionally, two samples à 1.5 ml are stored in sterile 2 ml cryo tubes (Sarstedt) in liquid nitrogen gas phase.

The PAXgene RNA tube is mixed ten times by turning after blood draw and is immediately centrifuged at 2,300 g for 10 min at 4 °C. From the supernatant, two aliquots with 1 ml each are transferred to sterile 2 ml cryo tubes (Sarstedt). The remaining supernatant is transferred to a fresh 15 ml tube (Sarstedt) without disturbing the pellet. Supernatant (1 ml) is used to resolve the pellet in the PAXgene tube and to transfer it to a sterile 2 ml cryo tube (Sarstedt). PAXgene samples are stored in liquid nitrogen gas phase.

Urine is sampled from midstream into a sterile 100 ml cup with a screw cap (Sarstedt). Within 15 min, two urine monovettes (Sarstedt) are filled and centrifuged at 2,300 g for 10 min at 4 °C. Afterwards, 1.5 ml supernatant is transferred to a sterile 2 ml cryo tube (Sarstedt) and stored in liquid nitrogen gas phase. The urine in monovettes is stored at -80 °C. During centrifugation, a Clinitek Microalbumin 9 reagent strip (Siemens Healthcare Diagnostics Inc., Tarrytown, NY, USA) is applied on the remaining urine in the cup for analysis of albumin, blood, creatinine, glucose, ketone, leukocytes, nitrite, protein, and pH.

### Information for study participants after the study program

Each participant who requests feedback from the results of his/her medical examinations and standard laboratory parameters obtains these in a written letter. If applicable, we suggest that the participant seeks advice from his/her general practitioner. In the case of severe abnormalities in the examinations (i.e., life-threatening and treatable), medical support is sought immediately, which is facilitated by our study center being located directly at the University Hospital Regensburg.

## Discussion

AugUR is a study platform with a focus on the elderly population from the city and county of Regensburg, Germany. It incorporates extensive biobanking, genotyping and a modern study protocol specifically tailored to the conditions of the elderly. Embedded sub-studies and measurements in biosamples using ‘–omics’ techniques will make this platform an important resource for research in the elderly.

What can be gained from in-depth epidemiological data in the elderly? A cross-sectional study with participants starting at 70 years of age is usually enriched with cases with a certain disease, and well-matched healthy controls are available. A longitudinal study design also allows for analyzing risk factors, such as the influence of life style factors on disease development and progression, and the interaction of non-genetic with genetic factors. The longitudinal aspect of a study with elderly participants can provide sufficient numbers of incident cases more quickly than a study with younger participants.

Beyond these major objectives of studying disease etiology, a study in the elderly has further practical usability: It yields *prevalence* and *incidence *estimates for late-onset diseases that are important determinants for public health managers. Such estimates require high-quality data and cannot be easily transferred across countries due to differences in health care systems, genetic background, climatic conditions, and life style. Additionally, a study in the elderly can also serve as a *control group* for case-control comparisons, which overcomes many of the usual challenges of control group identification: Control groups in genetic studies are often enriched for other diseases, e.g. by recruiting from hospitals, or are substantially younger or from a different region or even country than the patient group. Finally, *reference values* of clinical diagnostic parameters in the elderly are often lacking and warranted as most such parameters are evaluated in ‘normal’ adults, but applied to the patients entering the clinics – which are, mostly, of higher age.

We need to acknowledge a limitation of our study, which arises from the fact that only a certain proportion of invited persons will respond and participate and that the response will depend on health status: since all participants are required to make their appointment by phone and to come to the study center in-person, a certain level of mobility and hearing/cognitive ability is required that is not self-evident in that age range. Therefore, we will recruit from the ‘mobile’ portion of the general elderly population.

In summary, our population-based cohort study in the elderly is a platform that will help to improve our understanding of late-onset disease mechanisms that may, ultimately, lead to improved prevention, diagnosis, and therapy.

## Abbreviations

AugUR: *A*ge-related diseases: *u*nderstanding *g*enetic and non-genetic influences - a study at the *U*niversity of *R*egensburg
AMD: Age-related macular degeneration
GWAS: Genome-wide association study
CNV: Choroidal neovascularization
GA: Geographic atrophy
eCRF: Electronic case report form
ETDRS: Early treatment diabetic retinopathy
DRS: Digital retinography system
OCT: Optical coherence tomography
cSLO: Confocal scanning laser ophthalmoscopy
SD-OCT: Spectral-domain optical coherence tomography
ART: Automatic real-time
AGE: Advanced glycation end products
K3-EDTA: tripotassium ethylendiaminetetraacetate
